# Cyclosporin A triggers a RIG-I-mediated interferon response through multiple cellular processes

**DOI:** 10.1038/s41598-026-68563-x

**Published:** 2026-09-16

**Authors:** Lars Meier, Martin Schauflinger, Pauline Neubecker, Dennis Freisem, Lena Gimpl, Michelle Gellhorn Serra, Helene Hoenigsperger, Benjamin Rupf, Susanne Herold, Stefan Bauer, Konstantin M. J. Sparrer, Stephan Becker, Lucie Sauerhering

**Affiliations:** 1 Institute for Virology, Philipps-Universität Marburg, Marburg, Germany; 2 Philipps-Universität Marburg, Member of the German Center for Infection Research (DZIF), TTU Emerging Infections, Hans-Meerwein Straße 2, 35043 Marburg, Germany; 3 Institute of Molecular Virology, Ulm University Medical Center, Ulm, Germany; 4 Institute for Immunology, Philipps-Universität Marburg, Marburg, Germany; 5 Department of Medicine V, Internal Medicine, Infectious Diseases and Infection Control, Universities of Giessen and Marburg Lung Center (UGMLC), German Center for Lung Research (DZL), Giessen, Germany; 6 German Center for Infection Research (DZIF), Braunschweig, Germany; 7 Member of the Cardio-Pulmonary Institute (CPI), Frankfurt am Main, Germany; 8 Institute of Lung Health (ILH), Justus Liebig University Giessen, Giessen, Germany; 9 German Center for Neurodegenerative Diseases (DZNE), Ulm, Germany

**Keywords:** Cyclosporin A (CsA), Type III interferon, Mitochondria, Drug repurposing, Cell biology, Immunology, Microbiology

## Abstract

The development of broad-spectrum antiviral agents is essential for preparedness against emerging viruses with pandemic potential. Cyclosporin A (CsA), a clinically approved immunosuppressant, has been shown to exert antiviral activity against a wide range of viruses. Recent evidence suggests that this effect is partially mediated through the induction of interferon lambda (IFN-λ), which in turn stimulates a set of interferon-stimulated genes (ISGs) that establish an antiviral cellular state. However, the molecular mechanisms underlying IFN-λ induction by CsA have remained poorly understood. Here, we demonstrate that CsA triggers a retinoic acid-inducible gene I (RIG-I)-dependent activation of the IFN-λ/ISG signaling axis. This is accompanied by the upregulation of mitochondrial double-stranded RNA (dsRNA). Importantly, we show that CsA causes pronounced alterations in the mitochondrial cristae network. CsA also impairs autophagy, as evidenced by reduced autophagosomal membrane formation and increased levels of the autophagy marker p62. Supporting a functional link, treatment with the autophagy inducer Torin-1 reverses CsA-induced dsRNA accumulation, IFN-λ expression and ISG transcription. Together, our data indicate that the antiviral activity of CsA is partially driven by converging cellular processes involving mitochondrial remodeling, dsRNA accumulation, and impaired autophagy, which collectively activate the RIG-I/IFN-λ/ISG axis, offering mechanistic insights with therapeutic implications.

**Supplementary Information:**

The online version contains supplementary material available at https://doi.org/10.1038/s41598-026-68563-x.

## Introduction

Cyclosporin A (CsA) is an immunosuppressive drug, which has been used in post-organ transplantation therapy for decades^1,2^. CsA exerts its immunomodulatory effects on activated T-cells by forming a ternary complex with cyclophilin A (CypA) and the calcium-dependent phosphatase calcineurin^3^. Thereby the calcineurin-mediated dephosphorylation of the nuclear factor of activated T-cells (NFAT) is blocked^4–6^. Consequently, phosphorylated NFAT remains in the cytoplasm unable to translocate into the nucleus and initiate the transcription of cytokine genes. In addition to its immunosuppressive activity, CsA inhibits the C-Jun-N-terminal kinase (JNK)^7^.

Interestingly, CsA can also act antiviral inhibiting several viruses such *as* human immunodeficiency virus (HIV)^8^, hepatitis C virus (HCV)^9^ and Influenza A Virus (IAV)^10^. CsA affected incorporation of viral proteins into the virions (HIV) or into the viral replication complexes (HCV) or inhibited late stages of the replication cycle (IAV). Other studies, including our own, indicated that a CsA-mediated induction of the IRF1-dependent IFN-λ response in human lung epithelial cells played a pivotal role for the inhibition of coronaviruses in vitro, ex vivo and in vivo^11,12^. More precisely, we demonstrated that IFN-λ levels were indeed elevated in the bronchoalveolar lavage fluid of CsA-treated mice, indicating that this mechanism is functional in vivo. Importantly, these mice reached blood CsA concentrations comparable to those reported in patients receiving CsA treatment for transplantation^12,13^ providing evidence that these CsA levels are sufficient to induce IFN-λ responses and confer antiviral protection in vivo. The exact mechanism by which the IRF1/IFN-λ/ISG axis is activated after exposure to CsA remained elusive.

Type III interferons (IFN-λ1, IFN-λ2, IFN-λ3, and IFN-λ4) constitute a crucial first line of cellular defense against respiratory viral infections^14–16^. Their antiviral activity is mediated through the induction of a broad array of interferon-stimulated genes (ISGs), which act to restrict viral replication at multiple stages of the viral life cycle^17^. Initiation of IFN-λ induction involves the activation of cellular effectors, beginning with the detection of specific RNA signatures in the cytoplasm e.g., by retinoic acid inducible gene I (RIG-I)- like receptors (RLRs)^18^. Among other RNA species, RIG-I is activated by short double-stranded (ds) RNA molecules that are formed e.g., when viruses invade host cells and replicate their genomes. Upon activation, RIG-I initiates downstream signaling through its interaction with the mitochondrial antiviral-signaling protein (MAVS).

In addition to recognizing viral RNA, RIG-I is also capable of sensing endogenous dsRNA. For example, under oxidative stress, dsRNA can be generated within mitochondria and subsequently released into the cytoplasm^19,20^. To preserve cellular homeostasis, dysfunctional mitochondria and cytoplasmic dsRNA are typically removed via autophagy^21^. Impairment of these regulatory mechanisms can result in sustained RIG-I activation, aberrant induction of interferon signaling pathways, and the development of interferonopathies^21,22^.

Here, we provide data indicating that CsA modulates autophagy and alters mitochondrial morphology, which correlates with the accumulation of endogenous dsRNA. This CsA-induced dsRNA accumulation appears to activate a RIG-I–dependent signaling cascade, leading to the induction of IFN-λ and antiviral ISGs. The simultaneous impairment of autophagic processes by CsA may hinder the clearance of accumulated mitochondrial dsRNA in the cytoplasm and to a prolonged release of IFN-λ. Taken together, our findings suggest that CsA exerts antiviral activity through a dual mechanism: by activating innate immune responses via the RIG-I/IFN-λ/ISG axis, and by inhibiting cyclophilin-dependent viral processes as reported previously supporting its potential as a broad-spectrum antiviral agent.

## Results

### CsA initiates antiviral signaling dependent on RIG-I

**Fig. 1:**
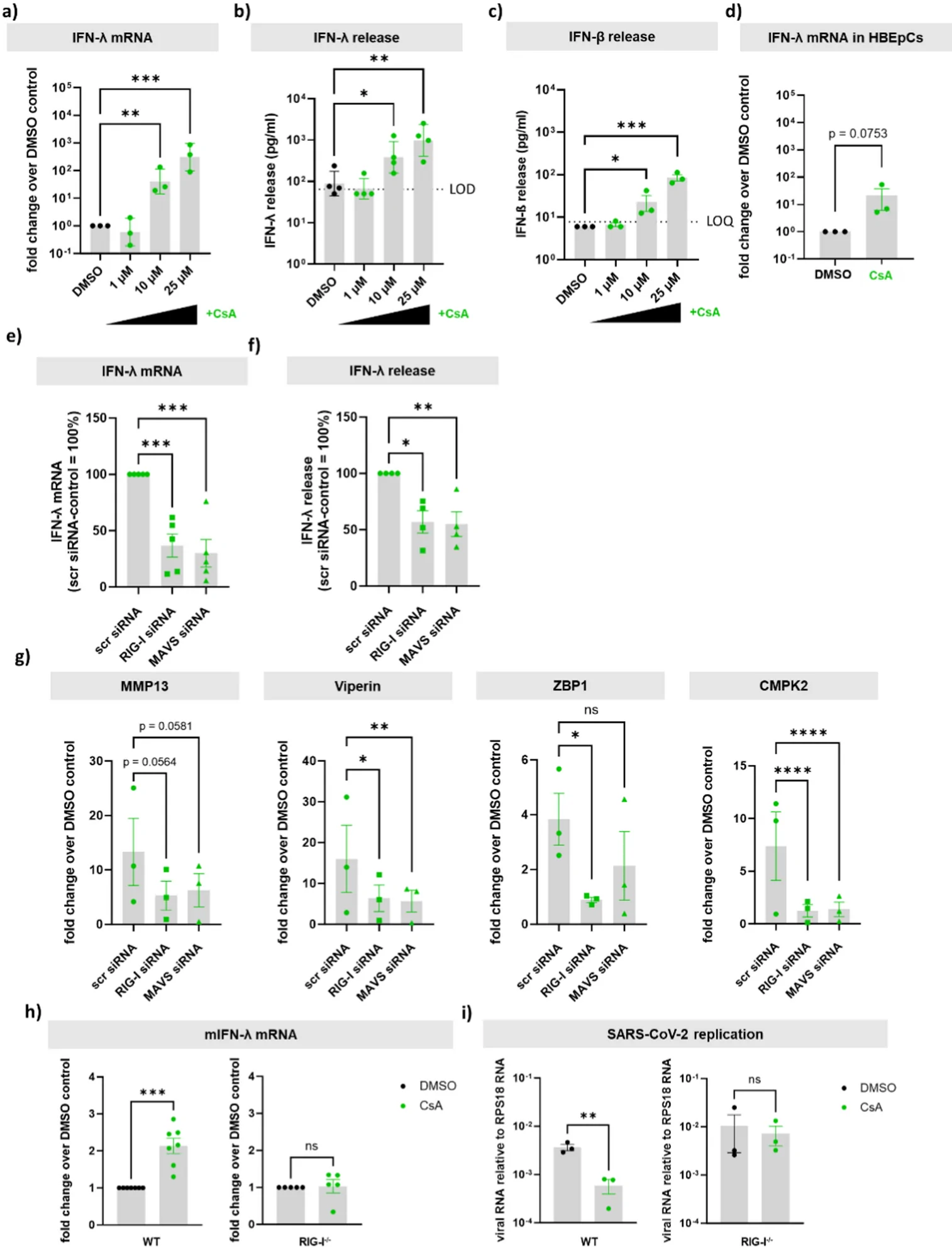
The RIG-I signaling pathway is essential for CsA mediated activation of IFNs and the antiviral effect. **(a)** Calu3 cells were treated with the indicated concentration of CsA or DMSO as solvent control. After 24 h RNA was isolated from cell lysates and analyzed for IFN-λ mRNA level. Data are represented as mean ± SEM of *n* = 3 independent biological replicates. **(b)** Released IFN-λ was measured in the supernatants of treated cells via IFN-λ 1/3 ELISA. Data points below the limit of detection (LOD) were defined as 50 pg/ml. Data are represented as mean ± SEM of *n* = 4 independent biological replicates. **(c)** Released IFN-β was measured in the supernatants of treated cells via IFN-β ELISA. Data points below the limit of quantification (LOQ) were defined as 6 pg/ml. Data are represented as mean ± SEM of *n* = 3 independent biological replicates. **(d)** Primary human bronchial epithelial cells (HBEpCs) were treated with 10 µM of CsA or DMSO. After 24 h RNA was isolated from cell lysates and analyzed for IFN-λ mRNA level. Data are represented as mean ± SEM of three biologically different donors. **(e)** Calu3 cells were transfected with specific siRNA for RIG-I or MAVS or scramble (scr) siRNA as control for 48 h. The medium was changed and the cells were treated with either CsA or DMSO as solvent control. After 24 h cells were harvested, RNA was isolated and qPCR analysis were performed for IFN-λ mRNA. Data are represented as mean ± SEM of *n* = 5 independent biological replicates. **(f)** The supernatant was analyzed for released IFN-λ by an IFN-λ 1/3 ELISA. (g) mRNA level of different ISGs were assessed in cells transfected with RIG-I or MAVS siRNA and stimulated with CsA (10 µM) for 24 h. Data are represented as mean ± SEM of *n* = 3 independent biological replicates. **(h)** mTBEpC isolated from WT or RIG-I KO mice were treated with either CsA or DMSO as solvent control. After 24 h the cells were harvested, RNA was isolated and qPCR was performed to detect murine IFN-λ. Data are represented as mean ± SEM of *n* = 5–7 independent biological replicates. **(i)** Isolated mTBEpC were transduced with a non-replicable adenovirus encoding for the human ACE2 at a MOI of 1000. 4 d later the cells were infected with SARS-CoV 2 using a MOI of 0.05 and after 24 h cells were harvested, RNA was isolated and qPCR for SARS-CoV 2 E RNA was performed. Data are represented as mean ± SEM of *n* = 3 independent biological replicates. Ordinary one-way ANOVA was used for statistical analyses comparing all groups to DMSO control **(a**,** b**,** c)** or scr siRNA control group **(e-g)** and for **g**) with experiment mean-normalized data. Unpaired t-test **(d**,** f**,** g)** were used comparing groups to DMSO control. *: *p* < 0.05; **: *p* < 0.01; ***: *p* < 0.005; ****: *p* < 0.001; ns or not indicated: not significant.

To assess the effect of cyclosporin A (CsA) on type III interferon induction, bronchial epithelial cells were treated with increasing concentrations of CsA. This resulted in a dose-dependent upregulation of IFN-λ mRNA expression, with significant induction observed at 10 µM and 25 µM, accompanied by increased IFN-λ secretion (Fig. 1a, b). Of note, the concentrations used were confirmed to be non-toxic (Fig. S1). In addition, we analyzed the release of IFN-β. A dose-dependent increase in IFN-β secretion was also observed (Fig. 1c), although absolute protein concentrations were approximately one order of magnitude lower than those of IFN-λ. This pattern is consistent with the well-established sequence of antiviral responses in epithelial cells, in which type III interferons are typically induced first, followed by a delayed and often weaker type I IFN response. Our findings are in line with this established paradigm and further support a predominant role of IFN-λ in the early antiviral defense of bronchial epithelial cells. Furthermore, in a previous study we could show that the antiviral effect of CsA is partially dependent on IFN-λ but not IFN-β^12^. Therefore, we focused on IFN-λ in the subsequent studies.

To validate the effect in a human-relevant context, we performed stimulation experiments using primary human bronchial epithelial cells isolated from three independent donors. Consistent with our findings, we observed CsA-mediated induction of IFN-λ (Fig. 1d) and ISGs (Fig. S2) in this physiologically relevant system. As previously reported, CsA-induced IFN-λ release requires activation and nuclear translocation of IRF1^12^. To investigate whether this process is mediated by the RIG-I/MAVS signaling axis, we performed siRNA-mediated knockdown of RIG-I and MAVS, followed by CsA stimulation. Silencing of either component led to a marked reduction in IFN-λ mRNA levels (Fig. 1e) and decreased IFN-λ secretion into the supernatant (Fig. 1f). Consistently, CsA-induced expression of several ISGs, including Viperin and CMPK2, was significantly reduced upon RIG-I or MAVS knockdown, while MMP13 and ZBP1 showed a similar trend with gene- and knockdown-dependent differences (Fig. 1g). These ISGs were previously identified among the most strongly upregulated genes following CsA treatment in Calu-3 cells^12^.

Moreover, we validated the involvement of RIG-I in CsA-mediated IFN-λ induction in primary tracheobronchial epithelial cells (mTBEpC) isolated from wild-type (WT) and RIG-I knockout (KO) mice. CsA treatment induced a significant increase in IFN-λ mRNA levels in WT cells, whereas this response was absent in RIG-I-deficient cells (Fig. 1h), highlighting the critical role of RIG-I in mediating the CsA-induced signaling cascade. To directly determine whether RIG-I is required for the antiviral activity of CsA, we infected mTBEpC from WT and RIG-I KO mice with SARS-CoV-2. In mTBEpC from WT mice, CsA effectively reduced SARS-CoV-2 infection (Fig. 1i), consistent with our previously published results regarding the inhibitory effect of CsA against SARS-CoV-2 and MERS-CoV in primary human bronchial cells^12,23^. In contrast, CsA showed no antiviral effect in mTBEpC from RIG-I KO mice (Fig. 1i). These results provide direct evidence that the initiation of antiviral pathways via the RIG-I/IFN-λ/ISG signaling axis is a critical component of CsA’s mechanism of action.

### CsA induces accumulation of mitochondrial dsRNA and changes in mitochondrial morphology

**Fig. 2:**
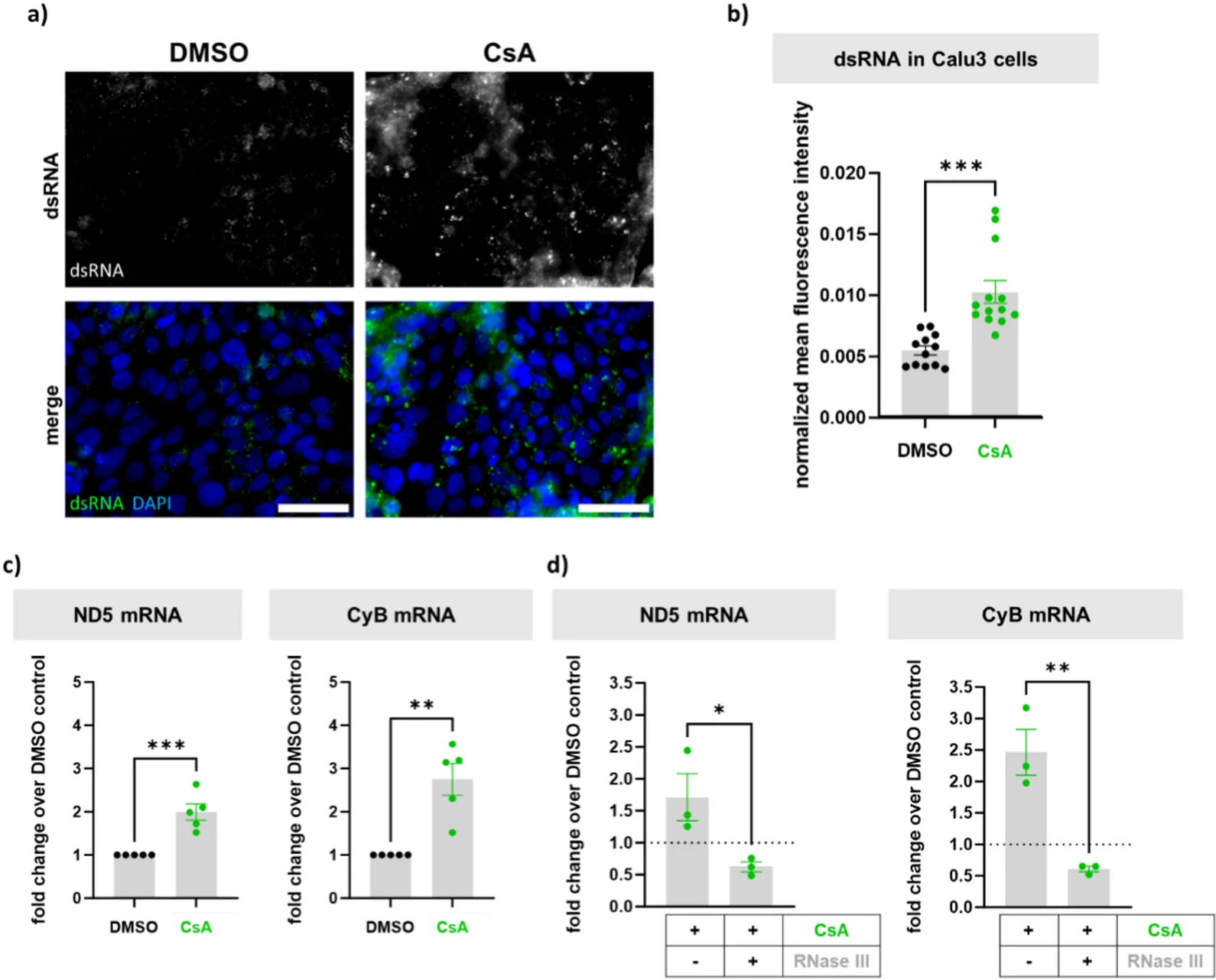
CsA induces mitochondrial dsRNA. **(a)** Calu3 cells were stimulated with 10 µM of cyclosporin A (CsA) or with DMSO as solvent control. After 24 h cells were fixed with 4% PFA for 1 h and dsRNA was stained with the anti-dsRNA antibody clone J2. Scale bar = 50 μm **(b)** The fluorescence signal for dsRNA was quantified using Fiji software and the calculated mean fluorescence intensities (MFI) were normalized to the MFI of the single experiments and cell count. Data are represented as mean ± SEM MFI from a total of 13 micrographs of *n* = 3 independent biological replicates. **(c)** Calu3 cells were treated with either CsA or DMSO as solvent control. After 24 h the cells were harvested, RNA was isolated and qPCR analysis were performed for different mitochondrial targets. Data are represented as mean ± SEM of *n* = 5 independent biological replicates. **(d)** For RNase III treatment the isolated RNA was incubated for 20 min at 37 °C with either 2U RNase III or dH2O as control (- RNase III treatment). The treated RNA was then analyzed by qPCR. Data are represented as mean ± SEM of *n* = 3 independent biological replicates. Unpaired t-test was used for statistical analysis. *: *p* < 0.05; **: *p* < 0.01; ***: *p* < 0.005.

RIG-I activation is initiated by the presence of cytoplasmic double-stranded RNA (dsRNA)^24^. To ascertain whether CsA treatment leads to dsRNA accumulation, we performed immunofluorescence analyses (IFA) in CsA-treated Calu-3 cells 24 h post-treatment. CsA stimulation resulted in marked cytoplasmic accumulation of dsRNA (Fig. 2a), and quantification of fluorescence intensity confirmed a significant increase in dsRNA levels (Fig. 2b).

Given that mitochondrial dysregulation is a known source of endogenous dsRNA^21^, we next analyzed the levels of two mitochondrial RNAs (ND5 and CyB) in CsA-treated Calu-3 cells at 24 h post-treatment. Both transcripts were significantly elevated upon CsA treatment (Fig. 2c). To determine whether the accumulated mitochondrial RNA was double-stranded, total purified RNA was treated with RNase III, which specifically degrades dsRNA^25^. This treatment abolished the CsA-induced increase in mitochondrial RNA, indicating that the observed signal corresponded to dsRNA (Fig. 2d).

**Fig. 3:**
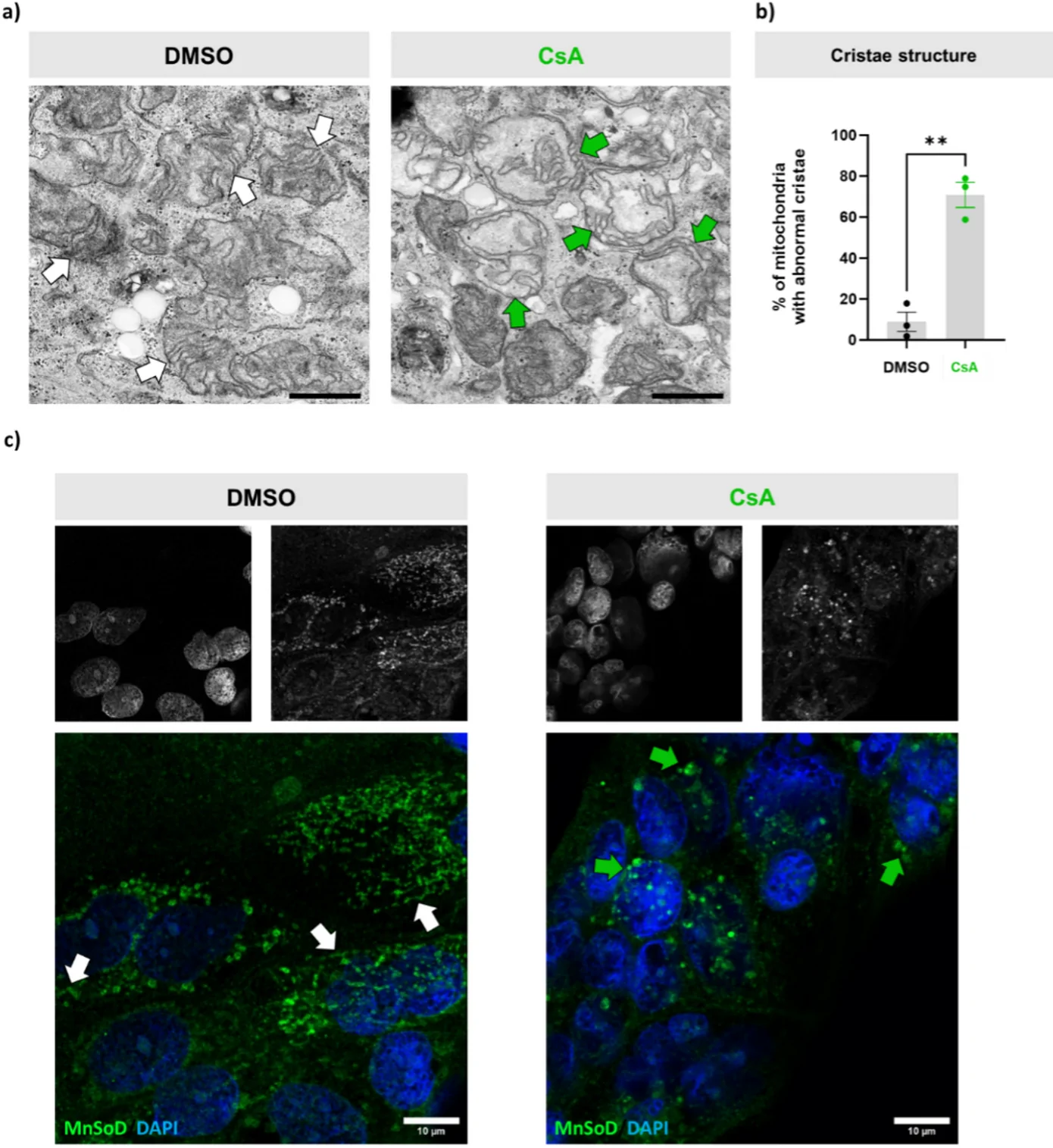
Mitochondrial morphology in CsA-treated cells. **(a)** Calu cells were treated with CsA (10 µM) or DMSO as solvent control. After 24 h the cells were fixed and processed for electron microscopy. White arrows indicate elongated mitochondria with stacked cristae. Green arrows indicate rounded mitochondria with disturbed, partially vesicularized cristae. Scale bars = 1 μm. **(b)** The fraction of mitochondria with abnormal cristae structure was determined by individually evaluating the mitochondria in micrographs of randomly selected cells. A total of 587 mitochondria from DMSO-treated cells and 383 from CsA-treated cells across 34 cells per condition from three independent biological replicates were evaluated. Data are represented as mean ± SEM from three biological replicates. **(c)** Calu 3 cells were seeded on cover slips and were stimulated with CsA (10 µM) or DMSO on the following day. After 24 h cells were fixed for 15 min with 4% PFA and stained with an α-manganese-dependent superoxide dismutase (MnSOD) antibody (1:50; Enzo Biochem). White arrows indicate elongated mitochondrial network, green arrows indicate roundish mitochondria. Scale bar = 10 µM. Unpaired t-test (b) was used for statistical analysis. **: *p* < 0.05.

To further investigate the impact of CsA on mitochondrial integrity, we conducted transmission electron microscopy (TEM) on Calu-3 cells treated with CsA or DMSO for 24 h. CsA-treated cells exhibited pronounced alterations in mitochondrial morphology, primarily characterized by changes in cristae structure (Fig. 3a and quantification in b). To confirm this result in another cell line, we also analyzed the effect of CsA on HeLa cells, which respond to CsA treatment with an induction of IFN-λ/ISGs signaling (Fig. S1a). In CsA-treated HeLa cells, TEM revealed similar remodulation of the mitochondrial cristae structure, which display either a vesicular or network-like appearance (Fig. S3b (green arrows) and quantification in c). In addition, distinct changes in mitochondrial size and shape were observed, reflected by altered circularity and area measurements (Fig. S3b and quantification in c). To further characterize the mitochondrial network, we performed IFA using the mitochondrial marker MnSOD (Manganese superoxide dismutase^26^. While DMSO-treated Calu-3 cells displayed (partially) a typical tubular network (indicated with white arrows), CsA-treated cells exhibited a disrupted, fragmented mitochondrial network (Fig. 3c). This phenotype is even more prominent in analyzed HeLa cells (Fig. S3e). Here morphometric analysis revealed a shift toward a more rounded mitochondrial shape, supported by increased circularity and decreased aspect ratio (Fig. S3f). These findings demonstrate that CsA induces significant alterations in mitochondrial morphology and network organization.

Whether these morphological changes directly contribute to the release of mitochondrial dsRNA remains unclear. However, the observed remodeling of mitochondrial structure in CsA-treated cells may facilitate or reflect the accumulation of cytoplasmic dsRNA.

### Cyclosporin A reduces autophagy and can be counteracted by the autophagy inducer Torin-1

**Fig. 4:**
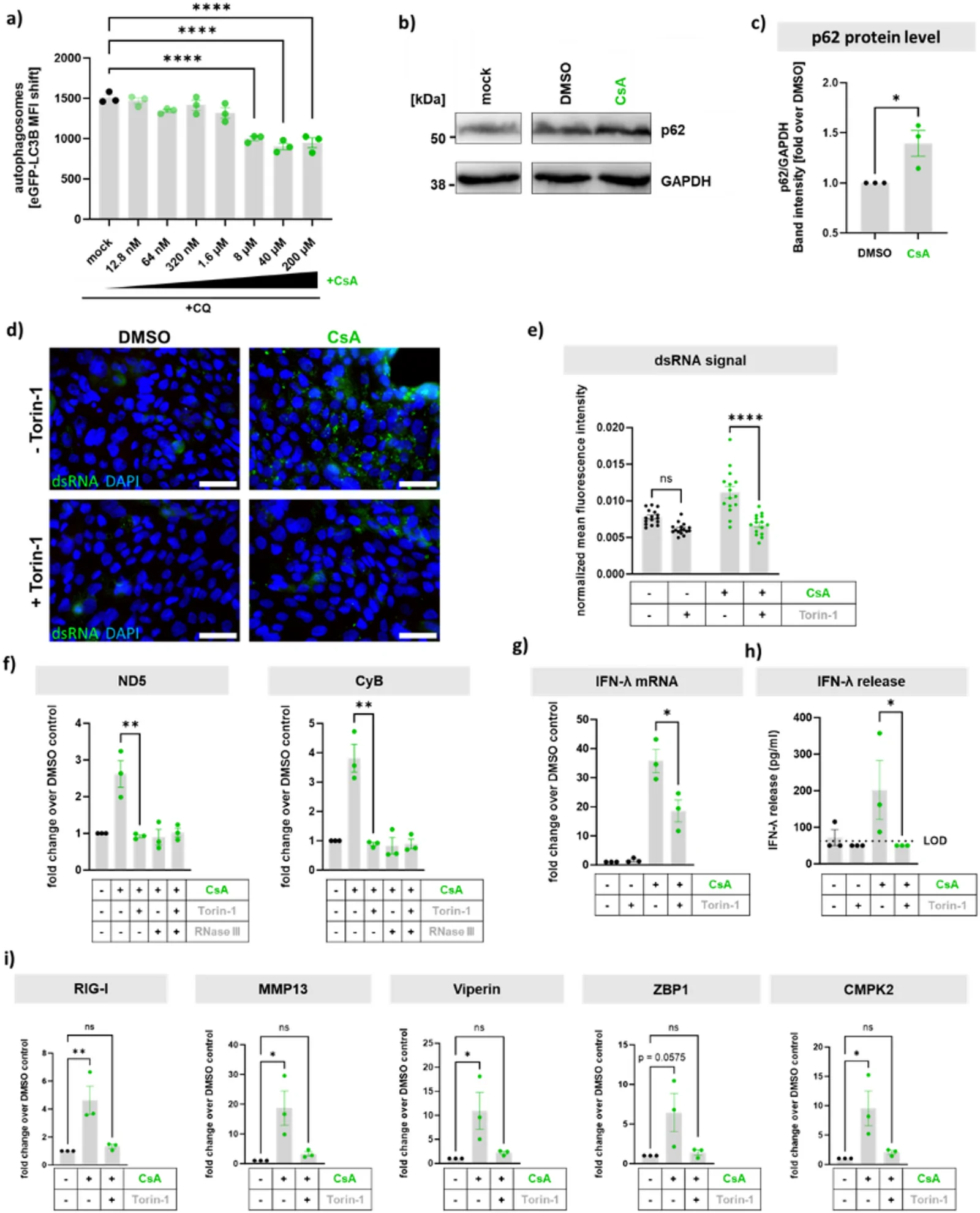
CsA treatment modulates autophagy and is counteracted by autophagy inducer Torin-1. **(a)** HeLa cells stably expressing eGFP-LC3B were stimulated with 20 µM Chloroquine (CQ) and the indicated concentrations of CsA for 4 h. The cells were analyzed via flow cytometry. The mean fluorescence intensity (MFI) of the mock control was subtracted from each sample. Data are represented as mean ± SEM of *n* = 3 independent biological replicates. **(b)** Calu3 cells were treated with 10 µM of CsA or DMSO as solvent control for 24 h. A representative immunoblot of whole cell lysates stained with anti-p62 and anti-GAPDH is shown. **(c)** Quantification of **(b)** using the Image Lab software. Data are represented as mean ± SEM of *n* = 3 independent biological replicates. **(d)** Calu3 cells were treated with DMSO or CsA (10 µM) and additionally with Torin-1 (1 µM) or DMSO. The cells were fixed after 24 h and stained for dsRNA. Scale bar = 50 μm. **(e)** The fluorescence signal for dsRNA was quantified by Fiji software and the calculated mean fluorescence intensities (MFI) were normalized to the MFI of the single experiments and cell count. Data are represented as mean ± SEM MFI of totally 15 micrographs of *n* = 3 independent biological experiments. Additionally, the RNA was isolated and analyzed via qPCR for mtRNA **(f)** and IFN-λ **(g)** mRNA levels, as well as ISG induction (i) and the supernatants were analyzed for released IFN-λ via ELISA **(h)**. Data points below the limit of detection were defined as 50 pg/ml. Data are represented as mean ± SEM of *n* = 3 independent biological replicates. **(f)** For RNase III treatment the isolated RNA was incubated for 20 min at 37 °C with either 2U RNase III or dH2O as control (- RNase III treatment). The treated RNA was then analyzed by qPCR. Data are represented as mean ± SEM of *n* = 3 independent biological replicates. Ordinary one-way ANOVA **(a; i)**, 2wayANOVA **(e)**, unpaired t-test **(c; f; g)** and unpaired t-test of log-transformed data **(h)** were used for statistical analyses. *: *p* < 0.05; **: *p* < 0.01; ****: *p* < 0.001; ns or not indicated: not significant.

To investigate the basis of increased dsRNA accumulation in CsA-treated cells which are degraded by autophagy under physiological conditions^21^, we analyzed key autophagy markers. First, we utilized HeLa cells stably expressing eGFP-LC3B^27,28^, which, upon activation of autophagy, is incorporated into autophagosomal membranes^29^. To induce autophagosome accumulation, we stimulated HeLa-eGFP-LC3B cells with chloroquine (CQ), an autophagic flux blocker resulting in a strong enhancement of the mean fluorescence intensity (MFI) (Fig. 4a, mock ≙ CQ only). Treating the recombinant HeLa cells additionally with increasing concentrations of CsA, dose-dependently reduced the amount of eGFP-LC3B-signal in the membranes (Fig. 4a), indicating that CsA can impede autophagosome formation, potentially leading to reduced degradation of dsRNA. To further support the impact of CsA treatment on autophagy, the autophagy marker p62 was assessed in Calu-3 cells (Fig. 4b). Here, a significant increase in p62 levels was observed in CsA-treated cells compared to the DMSO control (Fig. 4c). These findings are in line with in vitro and in vivo data from Li et al.^30^ and support the interpretation that CsA interferes with autophagy, which may contribute to the accumulation of endogenous dsRNA and subsequent initiation of the RIG-I-dependent IFN-λ signaling.

To support this hypothesis, we investigated whether the autophagy activator Torin-1 would reduce the CsA-mediated effects on different steps of the dsRNA-triggered RIG-I/IRF1/IFN-λ cascade in cells treated with both Torin-1 and CsA. The used concentrations of both compounds were not toxic to the cells (Fig. S3). Indeed, we observed a significant reduction in the level of dsRNA in CsA-stimulated cells after Torin-1 treatment, indicating that the induction of autophagy by Torin-1 can reduce the accumulation of dsRNA induced by CsA (Fig. 4d and quantification in e). In line with this, we detected less mtRNA in CsA-stimulated cells after Torin-1 treatment (Fig. 4f). RNase III-resistance of the mtRNA indicated that Torin-1 specifically counteracted dsRNA-induction mediated by CsA and effectively prevented CsA-induced dsRNA. Furthermore, we investigated the impact of Torin-1 on mRNA levels of IFN-λ and selected ISGs including RIG-I in CsA-stimulated cells. Treatment with Torin-1 resulted in a significant reduction of CsA-mediated IFN-λ mRNA and released protein amounts (Fig. 4g and h). Importantly, treatment with Torin-1 in the absence of CsA had no impact on IFN-λ mRNA level or release. In addition, mRNA levels of all analyzed ISGs were nearly restored to baseline levels by treatment with Torin-1, indicating that the autophagy inducer Torin-1 can counteract CsA-mediated IRF1/IFN/ISG signaling pathways (Fig. 4i).

In summary, the data presented here showed that CsA can induce robust IFN-λ by affecting autophagy and mitochondrial processes. This results in dsRNA accumulation and activation of RIG-I/IFN-λ/ISG pathways. Analysis of early timepoints revealed a distinct temporal sequence with first increased mtRNA levels (6 h p.s.), followed by IFN-λ secretion detectable at 12 h post-stimulation (as shown in^12^, and finally induction of ISGs at 18 h (Fig. S4). This ordered progression demonstrates a sequential activation of this pathway by CsA. In consequence, the cells reach an antiviral state, which could partially explain the broad-spectrum antiviral properties of CsA.

## Discussion

In our attempt to identify and characterize broad-spectrum antivirals we have characterized CsA which inhibits a number of RNA viruses. While some antiviral mechanisms of CsA seem to be highly specific, we have investigated a potentially broader effect, the induction of type III interferons. Importantly, the antiviral activity of CsA is likely multifactorial, combining direct effects on virus replication, including those mediated by cyclophilin inhibition, with indirect effects on innate antiviral immunity through IFN-λ induction. Our previous studies using neutralizing antibodies against IFN-λ further indicated that the antiviral activity of CsA is only partially dependent on IFN signaling, suggesting that IFN-independent mechanisms also contribute to its antiviral activity^12^.

During respiratory viral infection, the IRF1/IFN-λ/ISG pathway is induced upon recognition of viral dsRNA by various cytosolic RNA sensors, such as RIG-I or MDA5^31^. Although we do not demonstrate a direct activation of RIG-I in this study, we have shown that CsA triggered the RIG-I/MAVS-dependent signaling pathway similarly by inducing the cytoplasmic accumulation of endogenous dsRNA. Several endogenous RNA structures are capable of activating RIG-I. Those RNAs are formed during various physiological or pathological cellular processes. For example, the malfunction of RNA-targeting enzymes such as dual specificity phosphatase 11 (DUSP11) or the RNA editor adenosine deaminases acting on RNA (ADAR1) support the formation of RNA structures which can be recognized by RIG-I or MDA5 resulting in an IFN response independent of a viral trigger^32,33^. As shown by Dhir et al.^22^, most endogenous dsRNA are of mitochondrial origin. Furthermore, the potential of mitochondrial-derived dsRNA to induce inflammatory responses has been shown^19,22^.

The precise mechanisms underlying the accumulation of mitochondrial dsRNA and cytosolic dsRNA following CsA treatment remain to be elucidated. CsA is known to modulate mitochondrial function at multiple levels. For example, it inhibits the opening of the mitochondrial permeability transition pore (mPTP)^34,35^, an effect that has been reported to mitigate mitochondrial dysfunction in rat models of myocardial infarction. In our study, CsA treatment also altered mitochondrial morphology in a manner suggestive of inhibited fission activity. Mitochondrial dynamics are critically regulated by dynamin-related protein 1 (Drp1), which, in its active non-phosphorylated state, drives mitochondrial fission. The phosphorylation status of Drp1 is controlled by the phosphatase calcineurin^36^. As CsA inhibits calcineurin, CsA treatment could also lead to hyperphosphorylation of Drp1, thereby suppressing mitochondrial fission. Such a mechanism could account for the morphological changes in mitochondria observed following CsA treatment in our experiments. The observed morphological changes may be associated with alterations in mitochondrial integrity, potentially contributing to the cytosolic release of dsRNA, which was detected after CsA treatment of Calu-3 cells (Fig. 2). Under normal conditions, mitochondrial dsRNA released into the cytosol is eliminated via autophagy^21^, a process that appears to be impaired in CsA-treated cells.

Autophagy is a tightly regulated homeostatic mechanism^37^ that removes damaged or excess proteins, (ds)RNA and organelles through sequestration within double-membrane vesicles called autophagosomes, which subsequently fuse with lysosomes to degrade their cargo. This process is activated by the transcription factor EB (TFEB), whose activity is regulated by its phosphorylation status^38,39^. Phosphorylation by mammalian target of rapamycin (mTOR) retains TFEB in the cytosol, whereas dephosphorylation by calcineurin promotes its nuclear translocation and the transcription of autophagy-related genes^39–41^.

Inhibition of calcineurin by CsA, and the consequent maintenance of TFEB in its phosphorylated state, was shown to restrict TFEB’s translocation into the nucleus therefore repressing activation of autophagy-related gene expression^21,42^. The influence of CsA on TFEB phosphorylation was reported by Li and colleagues consistent with our hypothesis of CsA-mediated autophagy inhibition^30^. Consequently, the autophagy activator Torin-1, by inhibiting mTOR, counteracts CsA-mediated dsRNA accumulation (Fig. 4). In this context, it is noteworthy that CsA has been shown to prevent degradation of cytosolic mitochondrial proteins, suggesting that degradation pathways such as autophagy may be suppressed by CsA treatment. Consistently, Carreira et al. demonstrated that CsA inhibited autophagosome formation in starved rat cardiomyocytes^43^. Taken together, the presented data in the present study indicated that CsA blocks degradation of dsRNA by inhibiting autophagy. Importantly, those immunomodulatory properties were not attributable to any cytotoxic effects (Fig. S1).

**Fig. 5:**
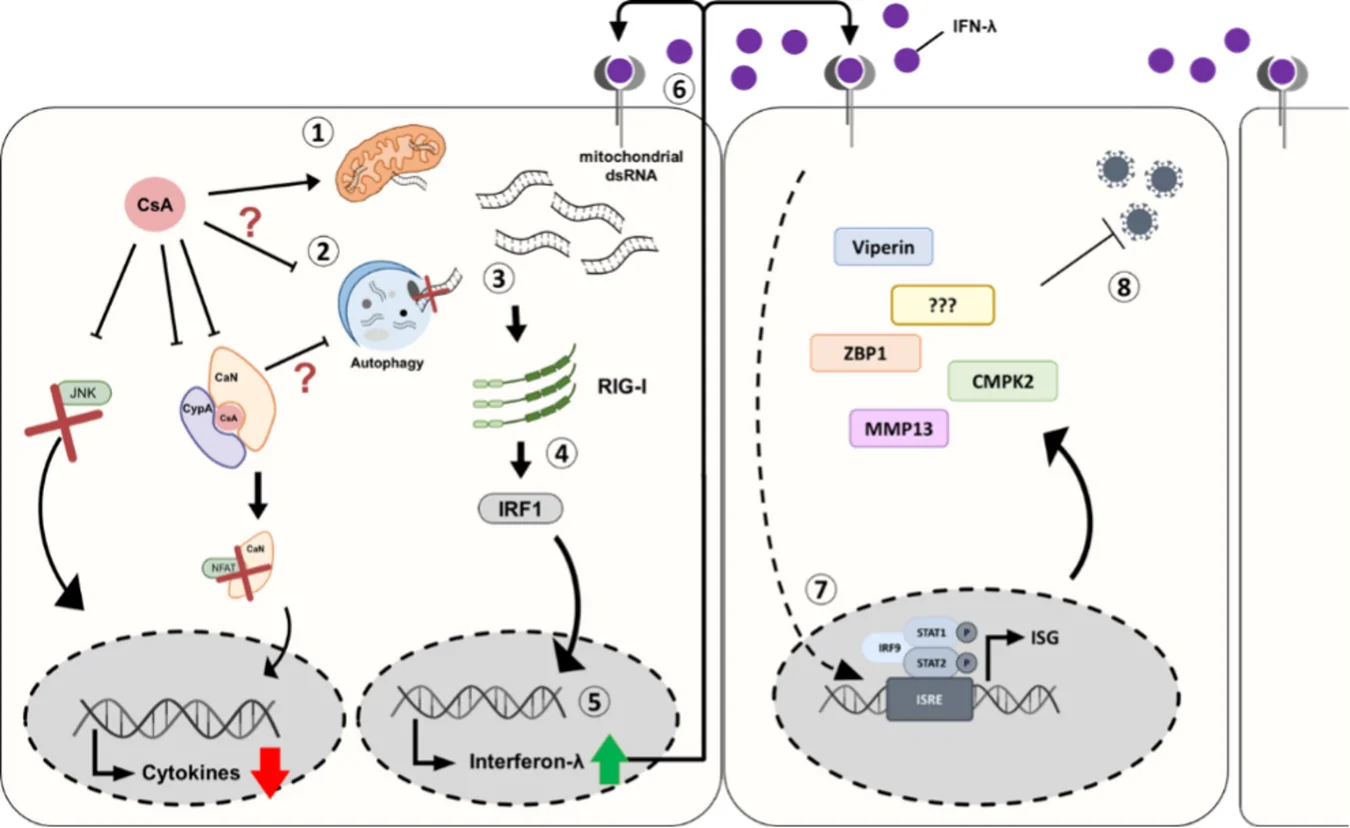
CsA activates an antiviral IFN response by influencing diverse cellular processes. CsA is known to inhibit the JNK pathway as well as CypA and Calcineurin (CaN). Furthermore, CsA leads to impaired mitochondrial morphology and integrity, as well as dsRNA in the cytosol and increased mtRNA^1^. In addition, CsA inhibits autophagy^2^, which possibly further increases the concentration of mitochondrial dsRNA in the cytoplasm^3^. Consequently, the accumulation of dsRNA activates e.g. RIG-I-dependent signaling pathways^4^ ultimately resulting in the induction and release of IFNs^5,6^ and the upregulation of interferon-stimulated genes^7^ mediating an antiviral state of treated cells^8^.

In summary, our findings indicate that CsA alters mitochondrial morphology and impairs autophagy, alongside the accumulation of RNase III-sensitive mitochondrial RNA and cytoplasmic dsRNA. These events are consistent with initiation of the RIG-I-mediated IFN-λ/ISG pathway and thus establishment of an antiviral state (Fig. 5). These results support the potential of CsA as a rapid-response therapeutic against newly emerging viruses by enhancing innate antiviral defenses in a virus-independent manner.

## Methods

### Cell culture

In this study, we utilized the human bronchial epithelial cell line Calu-3 and the cervix carcinoma cell line HeLa. Calu-3 cells were cultured in Dulbecco´s Modified Eagle Medium (DMEM)-F12 (1:1) supplemented with 1% Glutamine, 1% antibiotics and 10% Fetal Calf Serum (FCS, Gibco), while HeLa cells were cultured in DMEM with the same supplements. Primary human bronchial epithelial cells (HBEpC, Lonza) were cultivated using BEGM^®^ Bronchial Epithelial Cell Growth Medium (Lonza). All cell lines were maintained at 37 °C and 5% CO_2_.

### Inhibitor treatment

Stimulation experiments were performed using the cyclophilin inhibitor cyclosporin A (CsA, Sigma-Aldrich) at either a final concentration of 10 µM or a dilution series. As a solvent control, the cells were stimulated with the appropriate volume of DMSO.

To induce autophagy in cells, the autophagy inducer Torin-1 (InvivoGen) was added to freshly changed medium at a final concentration of 1 µM. To block autophagic flux for flow cytometric measurement, 20 µM chloroquine (Santa Cruz Biotechnology) were used.

### Cell viability assay

The CellTiter-Glo^®^ 2.0 Cell Viability Assay (Promega) was used according to the manufacturer’s instructions to analyze whether the used concentrations of CsA and Torin-1, as well as their combination, resulted in significant cell toxicity. Cells were seeded onto 96-well plates and treated with the respective compounds the following day. 24 h after treatment, 100 µl of CellTiter-Glo reagent was added to the cells and the cells were lysed by shaking the plate for 2 min. After 10 min incubation at room temperature, the luminescence was measured.

### Isolation of primary murine tracheobronchial epithelial cells (mTBEpC)

Wild type C57BL/6 mice were purchased from Charles River (Sulzfeld, Germany). RIG-I KO mice were generated and kindly provided by Stefan Bauer from the Institute for Immunology of the Philipps University of Marburg, Germany^44^. The isolation of primary tissues from mice was approved by the Animal Welfare Officer of Philipps University Marburg (Ex17-2021). Under German animal welfare legislation, a legal distinction is made between animal experiments and the killing of animals for scientific purposes. The procedures described in this study exclusively involved the killing of animals for scientific purposes in accordance with § 4^3^of the German Animal Welfare Act (TierSchG) and did not constitute animal experiments requiring authorization under German law. All procedures were performed in accordance with the relevant German guidelines and regulations. Male and female mice aged 2–12 months were randomly assigned to the experimental groups. TBEpC was isolated adapting the protocol published by Lam et al^45^.. Briefly, WT or RIG-I KO mice were narcotized by using 5% Isoflrane. After cervical dislocation, the thoracic cavity was opened and the trachea along with the primary bronchi were removed and transferred to a dish containing Ham´s F12 Medium (Pan Biotech). The surrounding tissue was removed and the trachea was opened lengthways followed by overnight incubation in 0,15% Pronae (Merck) in Ham´s F12 medium at 4 °C. On the next day, the detached cells were collected, centrifuged at 500 x g for 10 min and incubated with DNase (0.5 mg/ml; Sigma-Aldrich) in Ham´s F12 Medium for 5 min on ice. Finally, the cells were resuspended and seeded on Primaria™ tissue culture dishes from Corning™, which are suitable for primary cells. After 4 h, the supernatant containing unattached epithelial cells was collected and seeded onto plates, while the attached fibroblasts were discarded. The medium was changed on the next day and every other two days.

### Transduction of the primary murine cells

To ensure effective infection of mTBEpC with SARS-CoV-2, the cells were infected with a non-replicable Adenovirus that encodes for human ACE2 (ViraQuest Inc., North Liberty, IA, USA), the receptor for SARS-CoV-2. Cells were infected with a MOI of 1000 using serum-free medium as described by Sharma et al^46^.. After 1 h at 37 °C the inoculum was removed, the cells were washed with PBS_def_ and new medium was added. As described previously^47^, the cells were incubated for 4 days to achieve optimal ACE2 expression before being infected with SARS-CoV-2.

### Infection studies

Infections of hACE2-transduced mTBEpC with SARS-CoV-2 [BavPat1/2020 isolate, European Virus Archive Global 026 V-03883, München-1.1/2020/929] were conducted in the BSL-4 laboratory, Philipps-University Marburg, Germany. hACE2 expressing mTBEpC were infected with SARS-CoV-2 using a MOI of 0.05. Therefore, the supernatant was removed and the inoculum was added to the cells. Every 15 min the plate was shaken softly. After one hour of adsorption at 37 °C, the inoculum was removed and the cells were washed twice with PBS_def_. Finally, 1 ml of medium with 10% FCS was added and cells were incubated at 37 °C, 5% CO_2_ for 24 h.

### Immunofluorescence analysis

To analyze dsRNA or mitochondrial morphology, cells were fixed with 4% PFA for the indicated time at room temperature. Afterwards, the coverslips were washed three times with PBS_def_ and treated with 100 mM glycine in PBS_def_ for 10 min. 0.1% Triton X-100 in PBS_def_ was used to permeabilize the cells and any free binding sites were blocked by 2% BSA for 10–20 min depending on the used antibody. Finally, the mouse anti-dsRNA antibody clone J2 from Biozol (1:200) or a rabbit α-MnSOD antibody (1:50; Enzo Biochem) was incubated on the coverslips at 4 °C over night. A goat anti-mouse IgG or a goat anti-rabbit IgG antibody coupled with AlexaFluor 488 was used as the secondary antibody and incubated for 1 h at room temperature. The coverslips were washed three more times and then embedded on microscope slides. Imaging was performed using the fluorescence microscopy Axiovert200 from Zeiss or Stellaris 8 from Leica and was analyzed via the FIJI software (Version 1.53t) including the particle analysis to determine mitochondrial morphology.

### Quantitative real time PCR (qPCR)

Calu-3 cells and HeLa cells were lysed by using RLT-Buffer (RNeasy mini kit by QIAGEN) with beta-Mercaptoethanol (1:100). The supernatants were collected and the cell layer was incubated with 600 µl buffer for 10 min. Subsequently, the cells were scratched, transferred to a reaction tube and 600 µl of 70% ethanol was added. The RNA of the cell lysates was isolated using the RNeasy mini kit from QIAGEN following the manufacturer’s instructions.

The amount of RNA was measured by spectrometry utilizing the NanoDrop spectrophotometer by Thermo Fisher Scientific.

Using the LunaScript and the protocol recommended by NEB, 10.5 µl of total RNA was used for cDNA synthesis. For qPCR, an amount of cDNA equivalent to 50 ng RNA was used per reaction. The cDNA was mixed with water to a total volume of 9 µl per reaction. Furthermore, 10 µl of Luna master mix was mixed with 0.5 µl of forward and reverse primers (sequences in STable 1) and added to the sample. Each qPCR experiment was performed as triplicates of at least three individual biological experiments.

The delta-delta ct method (2^−ΔΔct^) was used to analyze the results for cellular mRNAs, which are depicted as fold change over control^12^. For analysis of viral RNA in infected cells and ISG mRNAs in primary cells, the amount of viral RNA/ISG mRNA is depicted in relation to the reference gene ribosomal protein S18 (RPS18).

### RNase III treatment and following qPCR

To analyze possible dsRNA in the previously isolated RNA, RNase III (by NEB) was added, which specifically degrades dsRNA. Therefore, the isolated RNA was divided into two parts. One part was treated with RNase III, while the other was treated with RNase-free water as a control. For both, 1 µg of the isolated RNA was mixed with RNase-free water to a total volume of 21 µl. Then, 3 µl of each 10x reaction buffer, RNase III or RNase-free water, respectively and MnCl2 (all delivered within the kit by the company) were added to the RNA. The samples were incubated for 20 min at 37 °C to allow degradation of potential dsRNA structures. Immediately after incubation, 7.5 µl of LunaScript master mix (NEB) was added and cDNA was synthesized.

For the following qPCR 1.875 µl of synthesized cDNA (corresponding to 50 ng RNA) was used for each reaction. β-actin was used as a reference gene in these experiments because it is known that RNase III can also cleave RPS18 RNA, likely due to its secondary structures^48^.

### IFN-λ ELISA

Supernatants of CsA treated and siRNA transfected cells were analyzed for released IFN-λ via ELISA. The commercial IFN-λ 1/3 ELISA kit from R&D was used following the manufacturer’s instructions. Briefly, the cell culture plates were coated with the primary antibody over night at room temperature. On the next day, free binding sites were blocked with reaction diluent for 2 h before 100 µl of the samples were added to each well and incubated for 2 h. Then, the plates were washed and 100 µl of the biotin-linked detection antibody were added and incubated for 2 h followed by a 20 min incubation with Streptavidin coupled to HRP. Between all steps, the wells were washed with supplied wash buffer. The enzyme reaction was started by adding 100 µl of the substrate solution and was stopped after 20 min by adding 50 µl of the stop solution. Finally, the absorbance was measured at 450 nm with 540 nm as reference measurement. The respective concentrations were calculated by using a standard curve.

###  IFN-ß ELISA

Released IFN-ß in supernatants of CsA-treated Calu-3 cells was quantified using the Human IFN-beta Quantikine ELISA Kit (R&D systems) according to the manufacturer’s protocol. Specifically, 50 µl of Assay Diluent RD1-19 were added to the IFN-ß pre-coated 96-well plate, followed by the addition of 50 µl of the samples or standards. After incubation for 2 h, the plate was washed four times. Subsequently, 200 µl of human IFN-ß conjugate were added to each well and incubated for additional 2 h. Upon completion of another washing step, 200 µl of substrate solution were added to each well and incubated for 30 min in absence of light. All incubation steps were performed at room temperature. Finally, the reaction was terminated by adding 50 µl of stop solution and the optical density was measured at 450 nm with wavelength correction at 540 nm, using a microplate reader. IFN-ß concentrations were calculated based on a standard curve.

### siRNA transfection

Knockdown studies were conducted using Silencer^®^ Select siRNA against RIG-I and MAVS, as well as Silencer^®^ Select Negative Control #2 siRNA (Thermo fisher scientific). Calu-3 cells were transfected with either specific silencer select siRNA or scramble silencer select RNA (scr siRNA) as a control at a cell confluency of 30–50% to analyze their role in the IFN-λ induction. The respective siRNAs and scramble siRNA were mixed with OptiMEM (Gibco) to reach a final concentration of 200 nM. The transfection reagent Oligofectamine was given to the siRNA and it was incubated for 20 min. The cells were washed with serum-free medium and 400 µl of the same medium was added. 100 µl of the siRNA/Oligofectamine mixture was slowly added to the cells. After 4 h, 250 µl of medium with 30% FCS was added to the cells to achieve a final FCS concentration of 10%. Finally, the cells were stimulated as previously described after 48 h.

### Quantification of autophagosomes

Quantification of autophagosomes was performed as previously described^49^. In brief, HeLa cells stably expressing eGFP-LC3B were treated with respective substances (CQ ± different concentrations of CsA) for 4 h. The cells were harvested using 0.05% typsin (PAN Biotech). After washing the cells once with PBS, they were permeabilized with 0.05% (/v) Saponin (Sigma-Aldrich) in PBS. Subsequently, the non-membrane bound eGFP-LC3B was washed out using PBS and afterwards cells were fixed in 4% (/v) paraformaldehyde in PBS. Quantification of the membrane-associated eGFP-LC3B, which reflects autophagic flux, was performed using flow cytometry (FACS Canto II, BD Biosciences). From each sample, the mean eGFP-LC3B fluorescence intensity (MFI) of the water control was subtracted.

### SDS-PAGE and western blot analysis

To generate whole cell lysates, medium was removed and cells were scraped off in 1 ml PBS. After centrifugation at 6000 rpm for 5 min, the cell pellet was lysed using cell lysis buffer (Thermo Fisher Scientific) supplemented with 1% protease inhibitor cocktail (Sigma-Aldrich) and incubated at ice for 30 min. The lysate was cleared by centrifugation at 4 °C, 14.000 rpm for 5 min and mixed with Tris-HCL buffered sample buffer containing 4% SDS, 10% β-Mercaptoethanol and 20% (v/v) Glycerol. Samples were boiled for 5 min at 95 °C and separated on a 15% SDS-PAGE at 90 V for 2.5 h. Proteins were blotted onto an activated PVDF membrane (pore size: 0,2 μm) at 30 V for 30 min. The membrane was blocked in 5% milk powder for 1 h before incubation with primary antibody (anti-p62, abcam, ab56416, 1:1000; anti-GAPDH, Thermo Fisher, AM4300, 1:4000)) in PBS-T (PBS with 0.1% Tween 20) with 5% milk powder (MP) overnight at 4 °C. The membrane was washed 3 times with PBS-T before incubation with 1:10,000 secondary antibody (HRP goat anti-Mouse IgG (Dako)) in PBS-T + MP for 1 h at RT. The membrane was washed 3 times with PBS-T and once with PBS. The SuperSignal Dura sustrate (Thermo Fisher Scientific) was used and the signal was measured at the CemiDoc imaging system.

### Transmission electron microscopy

For ultrastructural analyses, HeLa cells were seeded on Poly-L-Lysine coated sapphire discs (Engineering Office M. Wohlwend GmbH, Sennwald, Switzerland) one day before treatment with 10 µM CsA or DMSO. 24 h post treatment, the cells were fixed for 1 h in EM-grade 2.5% glutaraldehyde and 2% formaldehyde in 0.1 M Na-cacodylate buffer pH 7.2, followed by incubation in 1% osmium tetroxide containing 1.5% ferricyanide for 1 h in the same buffer. The cells were incubated in 2% uranyl acetate in water over night, dehydrated in a series of graded ethanol solutions into acetone, and subsequently infiltrated with resin (EMbed-812 Kit with BDMA), and polymerized at 60 °C for 48 h. The sapphire disc was removed to expose the cells at the blockface. Ultrathin sections were mounted on formvar-coated TEM grids, and double-stained with 2% uranyl acetate and 3% lead citrate. Samples were imaged at an acceleration voltage of 120 kV in a JEOL JEM-1400 TEM equipped with a TemCam-F416 camera (TVIPS, Gauting, Germany; unless otherwise mentioned, the materials are from Science Services, Munich, Germany). Characterization of mitochondria was performed on micrographs (magnification 5,000x) taken in randomly chosen cells from 3 biological replicates. To analyze mitochondrial shapes, individual mitochondria were manually traced using the Selection Brush Tool in ImageJ/Fiji. The outlines were added to the ROI Manager to measure area and shape descriptors. To analyze the mitochondrial cristae, the cristae network of each individual mitochondrion was evaluated for structural abnormalities. An abnormality was defined as the presence of vesicular cristae, i.e. cristae with a tubulo‑vesicular/saccular morphology as reported in electron microscopy studies (reviewed in^50^) including cristae with a network-like appearance. Statistical analyses were performed using GraphPad Prism.

*During preparation of this manuscript*,* the authors used DeepL Write*,* ChatAI and ChatGPT to improve readability. The authors subsequently reviewed and edited the text and take full responsibility for the content of the publication.*

## Supplementary Information

Below is the link to the electronic supplementary material.

Supplementary Material 1

## Data Availability

All data generated or analyzed during this study are included in this published article and/or its supplementary information files.
