# Supplementary material for: Cyclosporin A triggers a RIG-I-mediated interferon response through multiple cellular processes
Source: Sci Rep. 2026 Sep 16;16:28863. doi: 10.1038/s41598-026-68563-x (PMC13578290; doi:10.1038/s41598-026-68563-x)
Supplement: Supplementary file 1 [file 41598_2026_68563_MOESM1_ESM.pdf]

Supplement

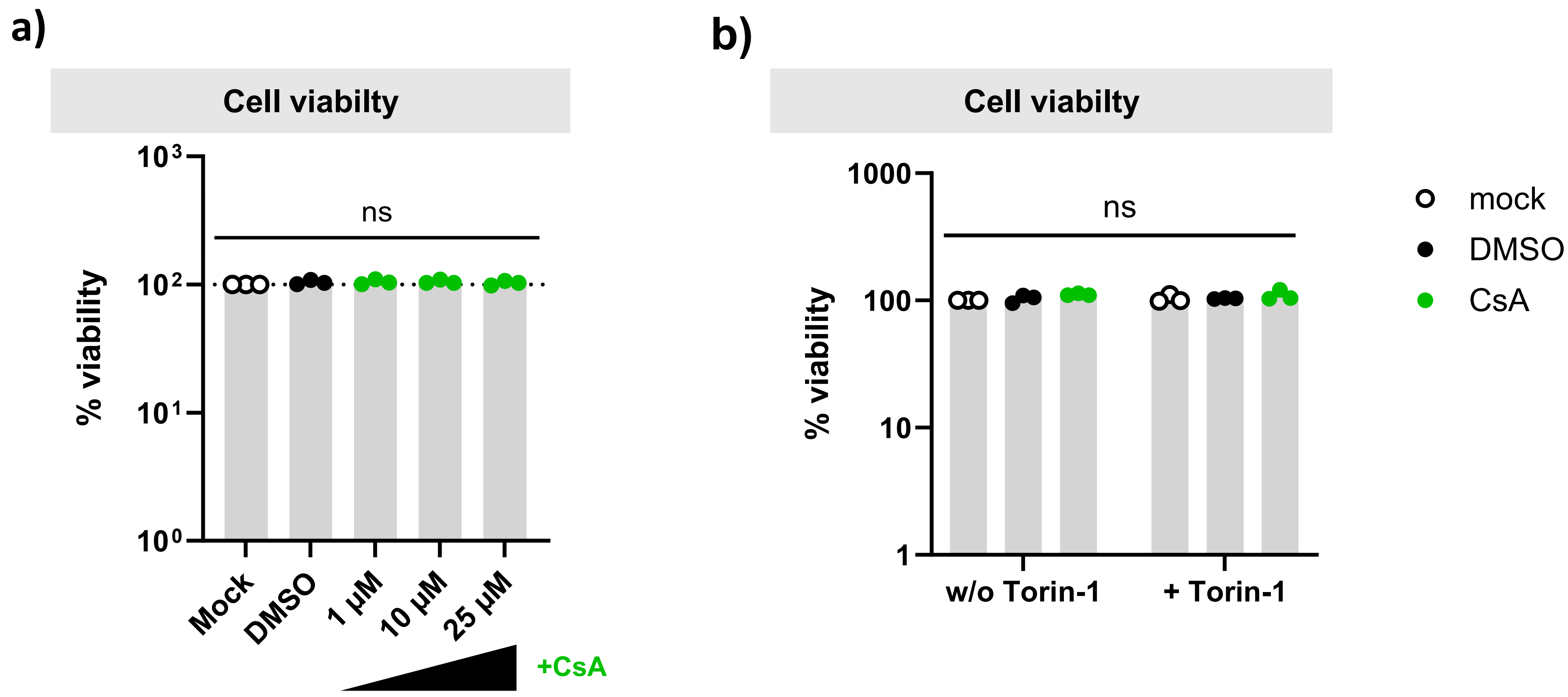

Supplement Figure 1: Cell viability in CsA and Torin-1 treated cells

(a) Calu3 cells were stimulated with DMSO or the indicated concentrations of CsA. After 24 h the cells were lysed and the amount of ATP was measured using the CellTiter-Glo® 2.0 Viability Assay (Promega). The values of the unstimulated cells were set to 100 %. Data are represented as mean ± SEM of n=3 independent biological replicates. (b) Calu3 cells were stimulated with DMSO or CsA (10 µM) alone or in combination with Torin-1 (1 µM). After 24 h the cells were lysed and the amount of ATP was measured using the CellTiter-Glo® 2.0 Viability Assay (Promega). The values of the unstimulated cells were set to 100 %. Data are represented as mean ± SEM of n=3 independent biological replicates. Ordinary one-way ANOVA (a) or One2wayANOVA (b) were used for statistical analyses. n.s: not significant.

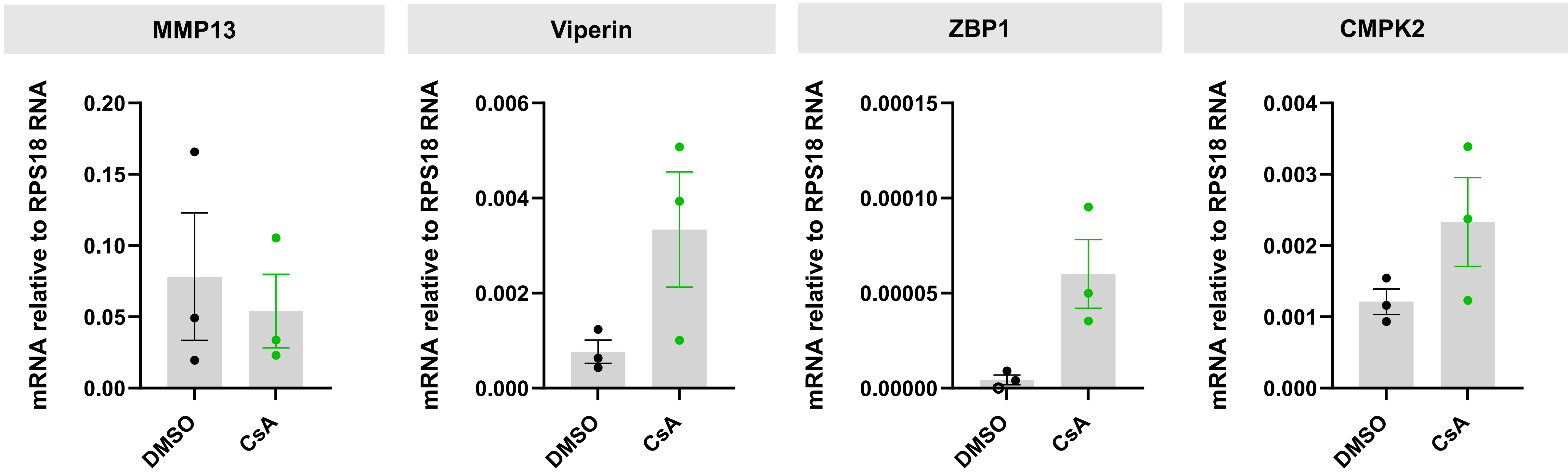

Supplement Figure 2: ISG induction in CsA treated primary human epithelial cells (HBEpC)

Primary human bronchial epithelial cells were treated with 10 µM of CsA or DMSO. After 24 h RNA was isolated from cell lysates and analyzed for mRNA level of the indicated ISGs. Data are represented as mean ± SEM of three biologically different donors. Data below the detection limit are represented by an empty circle.

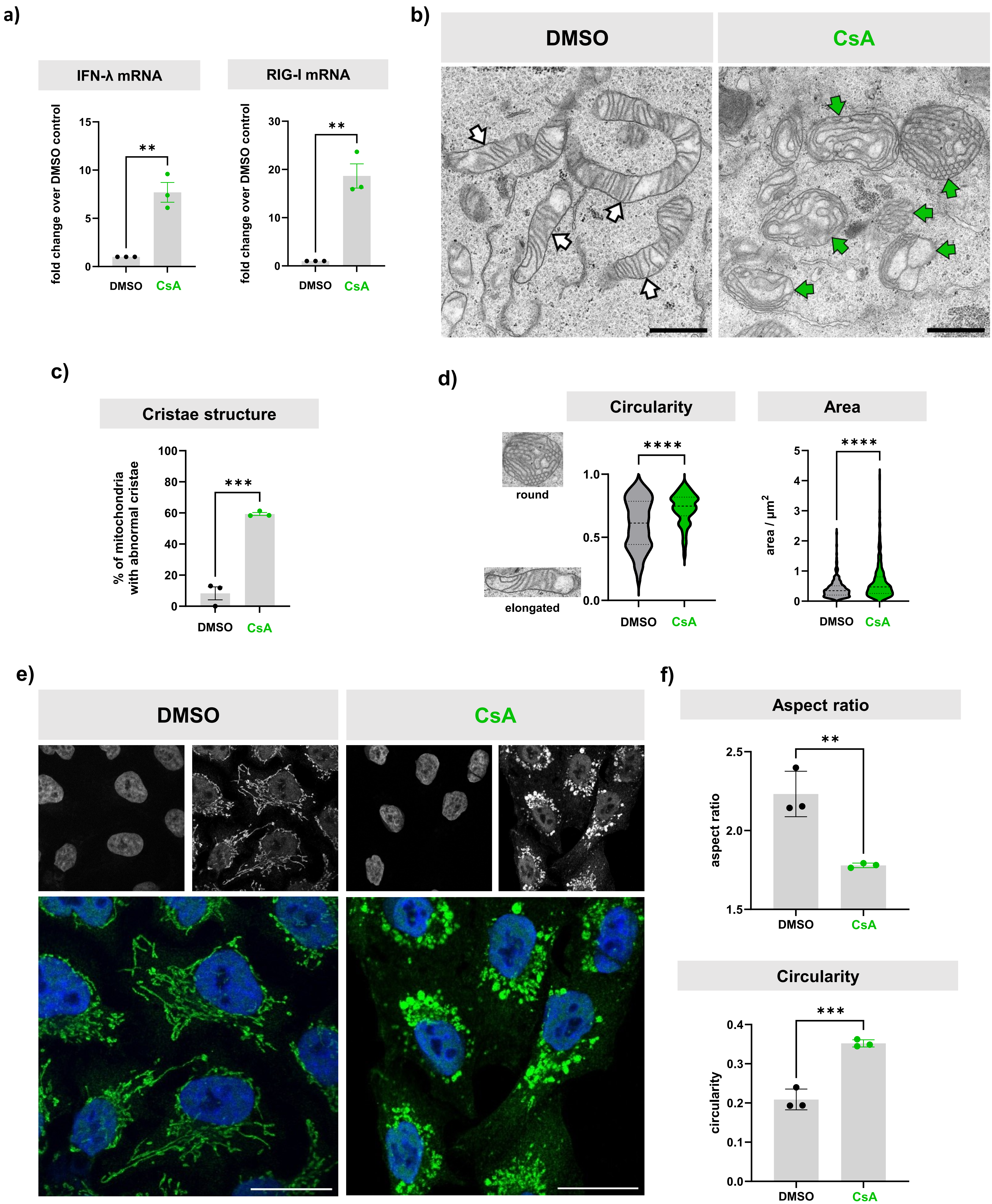

a)
IFN-λ mRNA
RIG-I mRNA
fold change over DMSO control
DMSO
CsA
b)
DMSO
CsA
c)
Cristae structure
% of mitochondria with abnormal cristae
DMSO
CsA
d)
Circularity
Area
round
elongated
area / µm²
DMSO
CsA
e)
DMSO
CsA
f)
Aspect ratio
aspect ratio
Circularity
circularity
DMSO
CsA

Supplement Figure 3: IFN activation and Mitochondrial morphology in CsA treated HeLa cells

(a) HeLa cells were stimulated with CsA (10 µM) or DMSO. After 24 h RNA was isolated from the cell lysates and analyzed for mRNA level of RIG-I and IFN-λ. Data are represented as mean ± SEM of three biologically different donors. (b) HeLa cells were treated with CsA (10 µM) or DMSO as solvent control. After 24h the cells were fixed and processed for electron microscopy. White arrows indicate mitochondria with stacked cristae. Green arrows indicate mitochondria with disturbed cristae structure. Scale bars 1µm. (c) The fraction of mitochondria with abnormal cristae structure was determined by individually evaluating the mitochondria in micrographs of randomly selected cells. We analyzed a total of 249 mitochondria from DMSO-treated cells and 231 from CsA-treated cells across 15 cells per condition from three independent biological replicates. Data are represented as mean ± SEM from three independent biological replicates. (d) The parameters of mitochondrial morphology were assessed by calculating the circularity as well as the area of the mitochondria using the ImageJ software. For the violin plots representing the morphology we analyzed a total of 249 mitochondria from DMSO-treated cells and 231 from CsA-treated cells across 15 cells per condition from three independent biological replicates. (e) Calu 3 cells were seeded on cover slips and were stimulated with CsA (10 µM) or DMSO on the following day. After 24 h cells were fixed for 15 min with 4 % PFA and stained with an α-manganese-dependent superoxide dismutase (MnSOD) antibody (1:50; Enzo Biochem). Scale bar = 20 µM. (f) Mitochondrial morphology parameters (aspect ratio and circularity) were assessed using the ImageJ software. Data are represented as mean ± SEM of three biologically different donors. Unpaired t-Test (a,c and f) and Mann Whitney test (d) was used for statistical analysis. **: $p<0.01$; ***: $p<0.005$; ****: $p<0.001$.

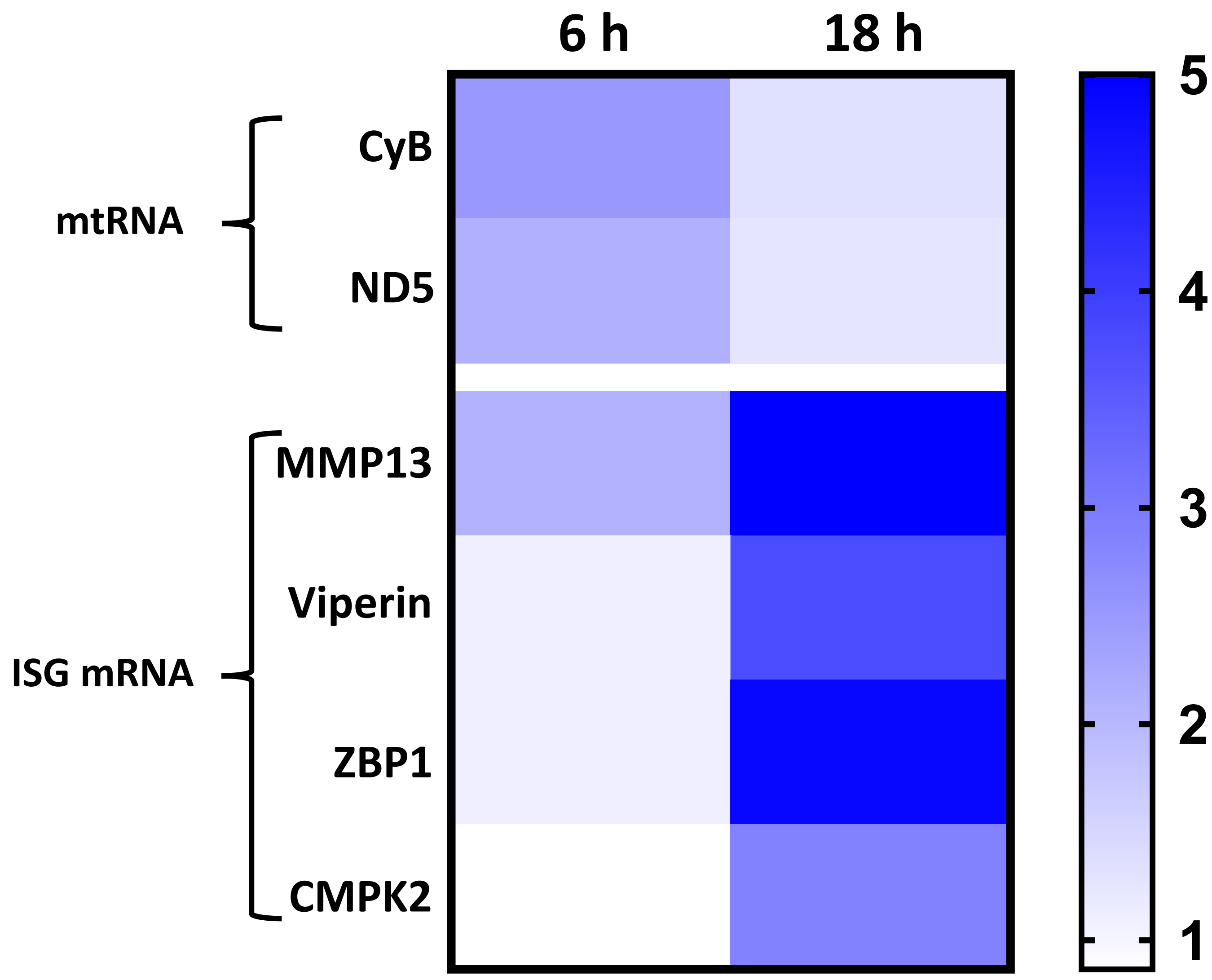

Supplement Figure 4: Early induction of mtRNA and ISG mRNA levels after CsA treatment

(a) Calu3 cells were stimulated with CsA (10 µM) or DMSO for 6 and 18 h. After the indicated timepoints RNA was isolated from the cell lysates and analyzed for mRNA level of CyB and ND5 (mtRNA) and MMP13, Viperin, ZBP1 and CMPK2 (ISGs). The heatmap represents the mean value of three independent biological replicates. Values reaching the upper limit of the scale were assigned a value of 5.

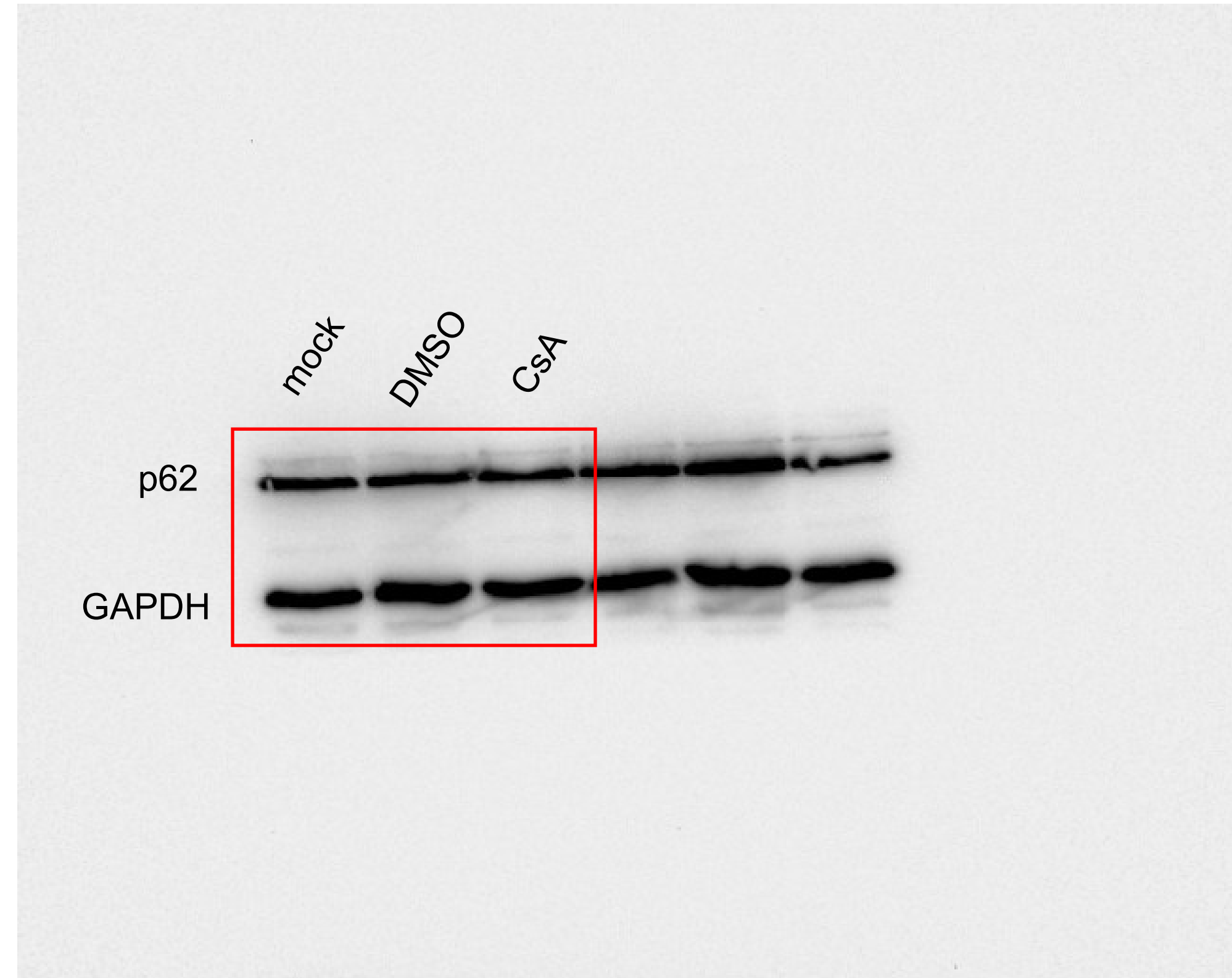

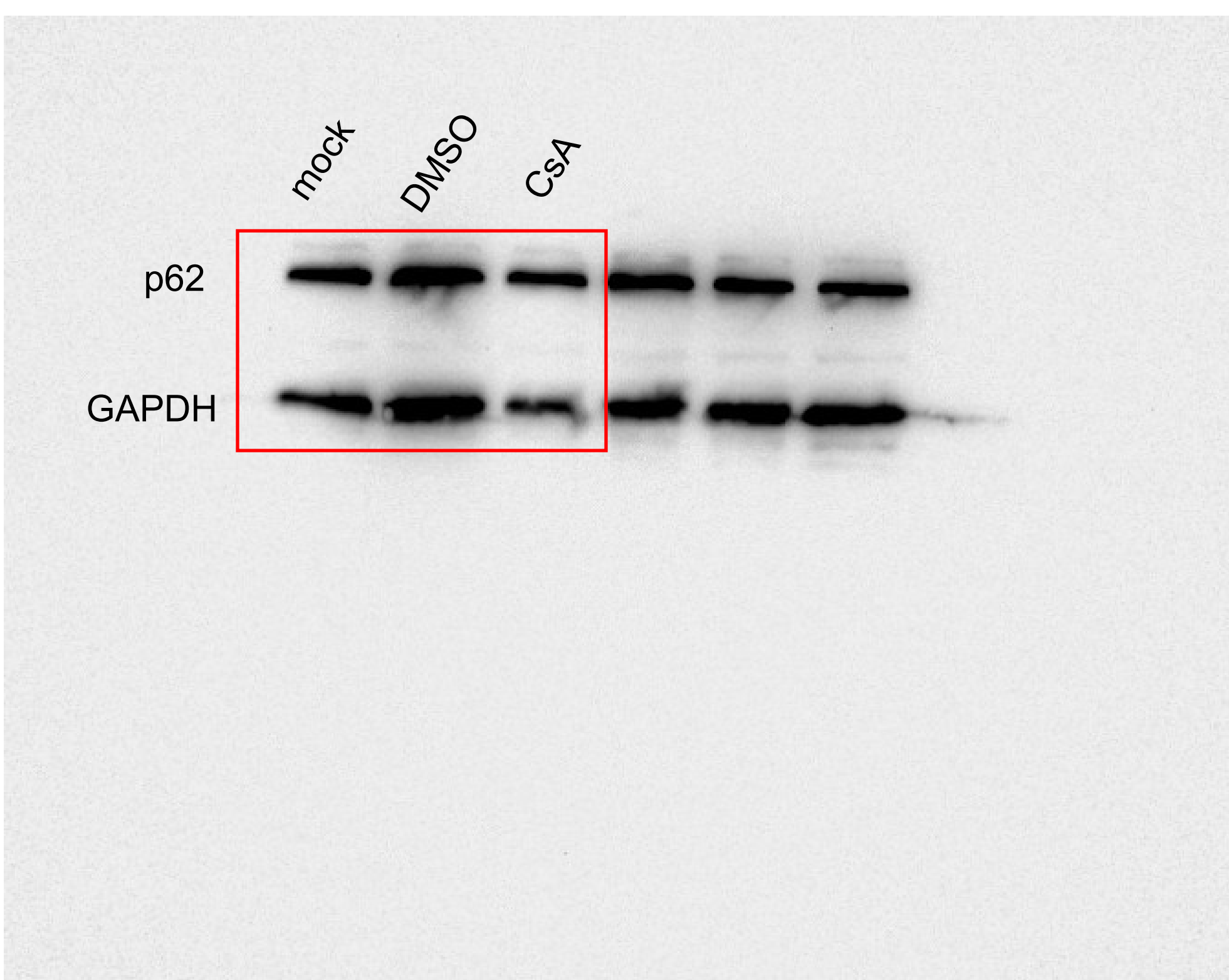

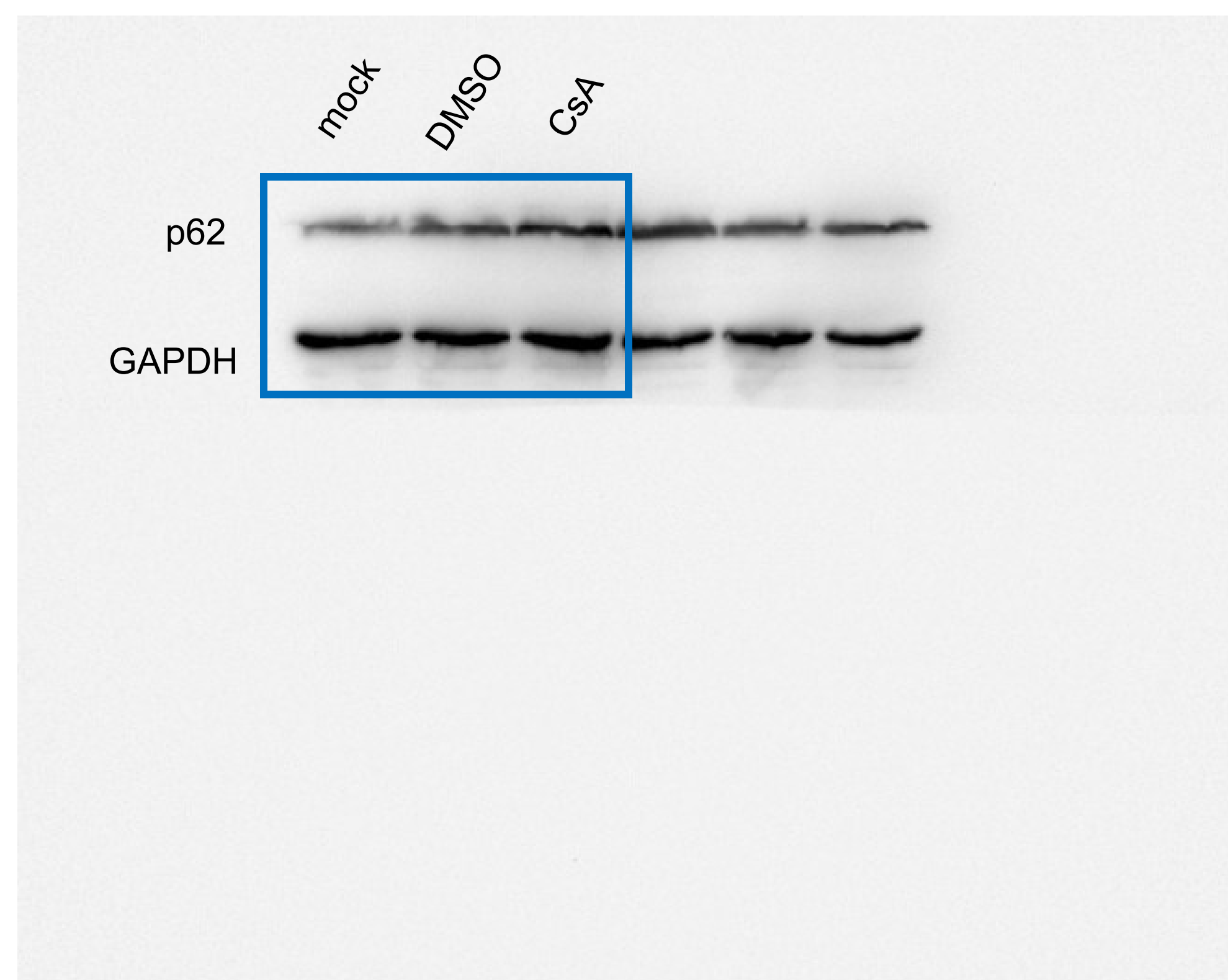

Supplement Figure 5: Uncropped blots of Fig. 4b and c
The uncropped blots are shown. Only the lanes highlighted by the boxes are presented in Figure 4b and c. The remaining lanes correspond to samples that were analyzed in the same experiment but are not included in the present study. The blue box indicates the results that are shown in Figure 4b).

Supplement Table 1: Sequences of the used RT qPCR primers

hCMPK2 forward	ACAGTGGATCTTGGAGTGGGA
hCMPK2 reverse	TCACCTGGTTCAACCTACTCCCT
hCyB forward	AGACAGTCCCACCCTCACAC
hCyB reverse	GGTGATTCCTAGGGGGTTGT
hβ-actin forward	CTGGGAGTGGGTGGAGGC
hβ-actin reverse	TCAACTGGTCTCAAGTCAGT
hIFNL 2/3 forward	GCCAAAGATGCCTTAGAAGAG
hIFNL 2/3 reverse	CAGAACCTTCAGCGTCAGG
hMMP13 forward	TCCCAGGAATTGGTGATAAA
hMMP13 reverse	CTGGCATGACGCGAACAATA
hND5 forward	TCGAAACCGCAAACATATCA
hND5 reverse	CAGGCGTTTAATGGGGTTTA
hRIG-I forward	GTGCAAAGCCTTGGCATGT
hRIG-I reverse	TGGCTTGGGATGTGGTCTACTC
hRPS18 forward	GCGGCGGAAAATAGCCTTTG
hRPS18 reverse	GATCACACGTTCCACCTCATC
hViperin forward	TGCCACAATGTGGGTGCTTACAC
hViperin reverse	CTCAAGGGGCAGCACAAAGGAT
hZBP1 forward	GCA AAC TCC GAA GCC ATC CAG A
hZBP1 reverse	CCA AGT TGA GGA ATC ACC TGG TG
mRPS18 forward	CCTGGATACCGCAGCTAGGA
mRPS18 reverse	GCGGCGCAATACGAATGCCCC
mIFNL 2/3 forward	AGCTGCAGGTCCAAGAGCG
mIFNL 2/3 reverse	GGTGGTCAGGGCTGAGTCATT
SARS-CoV 2 E forward	ACAGGTACGTTAATAGTTAATAGCGT
SARS-CoV 2 E reverse	ATATTGCAGCAGTACGCACACA
